# Transcriptional Changes of Cell Wall Organization Genes and Soluble Carbohydrate Alteration during Leaf Blade Development of Rice Seedlings

**DOI:** 10.3390/plants10050823

**Published:** 2021-04-21

**Authors:** Jae-Yeon Joo, Me-Sun Kim, Jwakyung Sung

**Affiliations:** Department of Crop Science, College of Agriculture, Life and Environment Sciences, Chungbuk National University, Cheongju 28644, Korea; kimms0121@chungbuk.ac.kr (M.-S.K.); jejeje2323@chungbuk.ac.kr (J.-Y.J.)

**Keywords:** cell wall, transcriptome, gene expression, soluble carbohydrates, rice

## Abstract

Plant cell walls have two constituent parts with different components and developmental stages. Much of the mystery concerning the mechanisms of synthesis, decomposition, modification, and so forth, has been resolved using omics and microscopic techniques. However, it still remains to be determined how cell wall development progresses over time after leaf emergence. Our focus in the present study was to expand our knowledge of the molecular mechanisms associated with cell wall synthesis in rice leaf blade during three distinct stages (sink, sink-to-source transition, and source). The RNA-seq, quantitative reverse transcription PCR (qRT-PCR) and carbohydrate concentrations were evaluated using developing fifth leaf blades harvested at different time points. The results revealed that some of the essential genes for the primary cell wall (PCW) were highly upregulated in the sink-to-source transition compared to the sink stage, whereas those essential to the secondary cell wall (SCW) displayed relatively higher levels (*p* < 0.05) during the source stage. The concentrations of soluble carbohydrates differed via type rather than stage; we observed higher monosaccharides during the sink stage and higher di- and oligo-saccharides during the sink-to-source transition and source stages. In conclusion, our findings suggest that the transcriptional regulation of plant cell wall biosynthesis genes are both synchronistic with and independent of, and directly and indirectly governed by, the abundance of soluble carbohydrates in the developing leaf blade, and, finally, raffinose is likely to play a transport role comparable to sucrose.

## 1. Introduction

The cell walls (CWs) are characterized into two types, primary and secondary. The formation of each CW is clearly distinguished by composition, developmental stage and function. Cellulose, xyloglucan (a hemicellulosic polysaccharide), pectin and lignin are known as principal components [1]. CWs play a variety of roles, including mechanical support, the regulation of cell-to-cell flow, and acting as a barrier against environmental stresses. CWs are usually affected in composition, thickness and strength through physicochemical and biological reactions during leaf development. These developmental processes are well regulated through a variety of functionally specialized enzymes, such as glycosyltransferases (i.e., cellulose-synthase-like, CslC), xyloglucan endotransglucosylase/hydrolase (XTH), glycosyltransferase (GT) and acyltransferase (AT). The primary cell walls (PCWs) and secondary cell walls (SCWs) are quite different in terms of composition, structure and function. PCWs which are typically defined as the network of cellulose microfibrils cross-linked by glycans, such as mannans, xylans, mixed-linkage glucans (MLG) and xyloglucans, are involved in defining cell shape and the resistance of tensile forces against turgor pressure [2,3]. By contrast, SCWs which are deposed after cessation of cell expansion and are made up not only of cellulose and hemicellulose but also lignin and cutin/suberin and provide mechanical support and protection against the biotic/abiotic stresses [4]. All plant leaves undergo both developmental stages, moving from the sink (net carbon importer) to the source (net carbon exporter). The growth of new leaves absolutely depends on the import of assimilates synthesized in older ones. The sink tissues, such as young leaves and roots, continuously receive photosynthetic products, mainly in the form of sucrose, until they can support themselves. In monocotyledonous plants, the sink and source stages simultaneously occur in the same tissue; the leaf tip matures as the leaf grows through the elongation zone at the base [5]. Therefore, during leaf development, the growth of the leaf blade is almost exclusively associated with leaf base elongation [6]. However, sink-to-source transition in the leaf blade continues until the blade reaches a specific developmental stage, which is characterized by the end of photo-assimilate import and the beginning of export [7]. The accumulation of soluble carbohydrates in leaves can have different effect on leaf development. For example, in the sink leaves soluble carbohydrates help with the construction of the photosynthetic system and cell compartments, on the other hand, higher levels of sucrose and sugar phosphate induce the feed-back inhibition of photosynthesis [8,9]. The concentrations of soluble carbohydrates in developing leaves differs with regard to the type of sugar, with increases in sucrose (disaccharide) being leaf aging-dependent and the highest levels of glucose, fructose, and galactose (monosaccharide) found in the elongation stage [10].

In this study, we aimed to gain a better understanding of CWs’ synthesis-associated molecular mechanisms in rice leaf blade during the three distinct stages of leaf development. To complete this task, we evaluated the temporal changes in transcriptional abundances and the soluble carbohydrates in developing rice leaf blades. Our data indicate a dynamic transcriptional process during leaf blade maturation. We have specifically described temporal differences in the transcriptome, which are associated with cell wall developmental metabolism.

## 2. Results

### 2.1. Leaf Blade Transcriptome and DEGs Analysis

**Figure 1:**
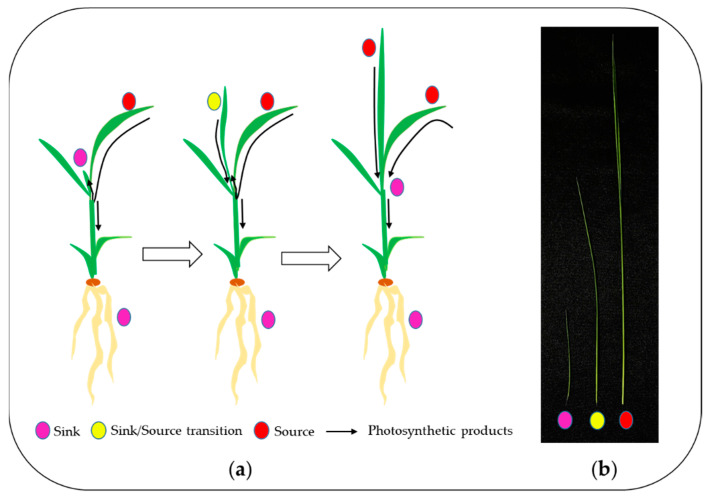
Transition process from sink to source tissue in rice seedlings (**a**) and 5th leaf blade at three different stages (**b**) (magenta, Day 1; yellow, Day 3; red, Day 5).

**Figure 2:**
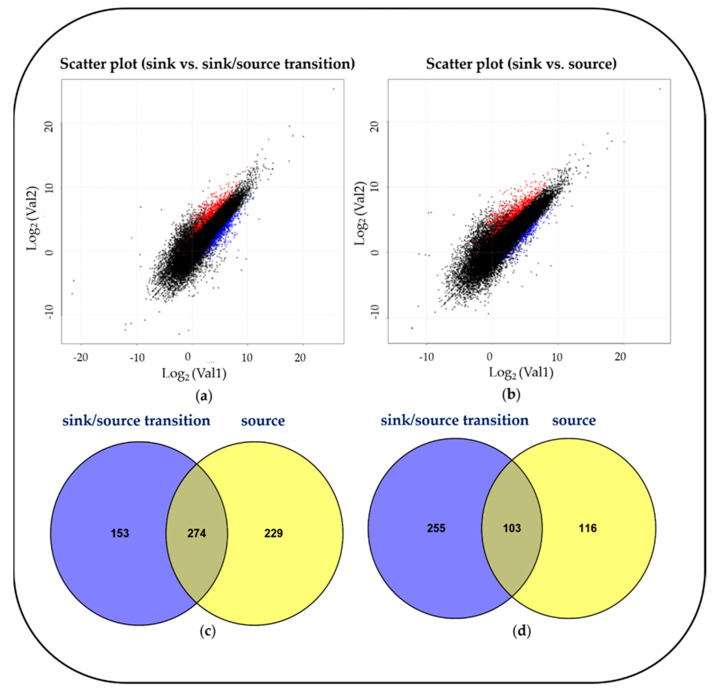
Scatter plot showing correlation between expressed genes. (**a**) Sink vs. sink-to-source transition. (**b**) Sink vs. source. Red spots, log_2_ fold change >1 and *p*-value < 0.05; blue spots log_2_ fold change < -1 and *p*-value < 0.05. Venn diagram showing the overlapping (**c**) upregulated and (**d**) downregulated differentially expressed genes (DEGs) in sink-to-source transition or source compared to sink.

**Figure 3:**
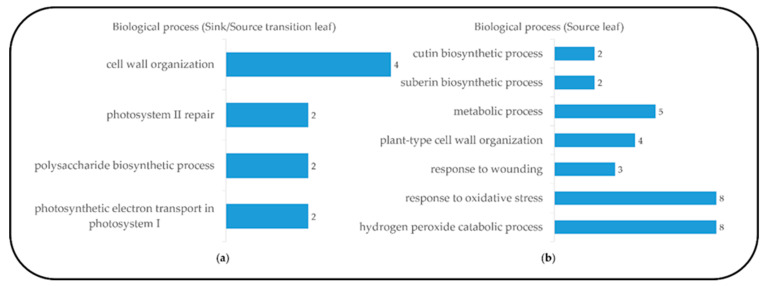
Functional category distribution of selected upregulated genes assigned by gene ontology (GO) terms to biological processes during sink-to-source transition and source stage in the blade tissue, from the database for annotation, visualization and integrated discovery (DAVID, v 6.8), sink-to-source stage (**a**) and source stage (**b**).

To investigate developmental changes in the transcriptome and metabolites in the blades, we harvested the blades of the fifth leaves, as follows: sink (Day 1, emergence (<0.5 cm) from the fourth leaf ligule), sink-to-source (Day 3) and source (Day 5) (Figure 1). The emergence of a new leaf usually takes place every five to six days [11], and an average time required for sink-to-source transition is 2.4 d [5]. From the RNA-seq analysis, we detected 26,750, 28,963 and 30,274 expressed genes from the sink, sink-to-source and source stages, respectively. A high number of differentially expressed genes (DEGs) from the blades were derived between two libraries, sink-to-source vs. sink and source vs. sink. A total of 3599 DEGs were identified using Cufflinks from two libraries, using the absolute value of fold change (log_2_ FC > 1 or log_2_ FC < –1) and statistical significance (*p* value < 0.05) for each gene. The transcriptome dynamics were visualized using MA plots in both libraries, sink-to-source vs. sink (Figure 2a) and source vs. sink (Figure 2b). The number of DEGs remaining after the removal of redundant genes was 1130, which were divided into 656 upregulated (Figure 2c) and 474 downregulated (Figure 2d) genes. To understand the biological functions of the identified DEGs, gene ontology (GO) functional enrichment analysis was performed on the sink-to-source (Figure 3a) and source (Figure 3b) genes, with both types showing upregulated expression. GO classification revealed the greatest abundance of genes encoding proteins involved in “autotrophy (photosynthesis) and structural development (cell wall)” during sink-to-source transition, and those encoding proteins involved in “structural integrity (cell wall), metabolism and environment-derived responses” during the source stage compared to the sink stage (Figure 3).

### 2.2. Validation of RNA-seq Data by qRT-PCR Analysis

To validate the RNA-seq data using real-time qRT-PCR, cell wall organization-related genes from groups of 153 (sink-to-source) and 229 (source) DEGs were selected. On the basis of GO analysis, we divided these genes into two categories, primary (sink-to-source transition) and secondary cell wall organization (source stage) related genes. The expression patterns of the selected cell wall development-related genes, namely, glycosyltransferase (GT 43), endotransglucosylase/hydrolase (XTH), Endoglucanase 16, cellulose-synthase-like C (CslC), glycosyltransferase (GT 8), fatty acyl reductase (FAR), ATP-binding cassette (ABC) transporter, glycerol-3-P acyltransferase (GPAT 3) and BAHD acyltransferase and peroxidase (POD), were positively correlated with the results of qRT-PCR (Table 1 and Table 2).

### 2.3. Primary and Secondary Cell Wall Organization and Soluble Carbohydrates Compositions during Leaf Development

**Figure 4:**
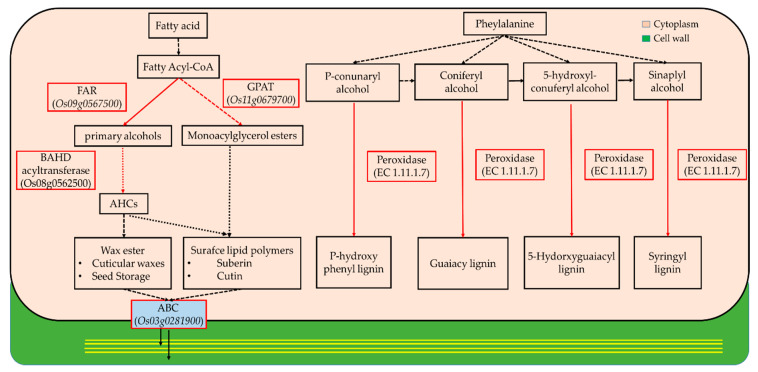
Simplified coordination process for secondary cell wall organization in plants. In the present study, genes in red boxes were highly upregulated during the source stage in the blades. FAR (fatty acyl-CoA reductase); GPAT (glycerol-3-phosphate acyltransferase); BAHD (benzyl alcohol *O*-acetyltransferase, anthocyanin *O*-hydroxycinnamoyltransferase, N-hydroxycinnamoly/benzoyltransferase and deacetylvindolin 4-*O*-acetyltransferase); ABC (ATP-binding cassette).

Plant cell walls, which are synthesized via two sequential steps, play a fundamental role in shaping and strengthening cells. We discovered that the expressions of cell wall-related genes during leaf development underwent changes, and these genes significantly differed depending on the stage (Table 1 and Table 2). As represented in Table 1, the markedly upregulated genes in sink-to-source transition exhibited GT 43, XTH, Endoglucanase 16, CslC and GT 8, which are used for primary cell wall organization as previously reported in grasses including rice [12,13,14,15]. The higher expression of these genes indicates that the primary cell wall is assembled by the coordinated decomposition, biosynthesis and remodeling of xyloglucan and xylan. In the source stage, FAR, ABC transporter, GPAT 3 and BAHD acyltransferase (for suberin/cutin biosynthesis), and POD (for hydrogen peroxide metabolism), displayed the highest expression levels (Table 2). The organization of the secondary cell wall was simultaneously implemented through the synthetic pathways of fatty acid-derived suberin and cutin and phenylalanine-derived lignin and, moreover, this process seemed to be dominant as the blades develop into source tissue (Figure 4). This explains that the organization of the primary and secondary cell walls is a process carried out in distinct time stages. To assess the stage-dependent changes in soluble carbohydrates in terms of substrate supply for cellular metabolism, their relative levels in two successive stages, compared to the sink stage, were measured (Table 3). The levels of monosaccharides were substantially reduced during both blade developmental stages, whereas di- (sucrose) and oligo-saccharides (raffinose) constantly increased throughout the period. The distinct stages specific to blade development included the production, conversion and allocation of soluble carbohydrates.

## 3. Discussion

The process of sink-to-source transition in developing leaf blades requires continuous photo-assimilate import until the blades reach 50%–60% of their final length, and this is the stage during which the blades elongate from 2 to 10 mm from the base [5]. In this study, we have investigated cell wall organization according to the transcriptomes present during rice leaf blade development. To acquire an abundant data set, we examined rice transcriptomics data from the different deposition stages of the leaf blades (sink, stage 1; sink-to-source, stage 2; source, stage 3). By comparing DEGs and identifying highly expressed genes during blade maturation (Figure 2), we found that the type of increased genes was strongly dependent on the maturity of the blades (Figure 3). We also found that the genes expressed in the blades during sink-to-source transition were involved in primary cell wall organization and photosynthesis, and in those expressed in the source stage were involved in secondary cell wall development and environmental responses. Interestingly, several genes were highly expressed, such as CslC, GT 8, GT 43, endoglucanase 16 and XTH. The functions of the enzymes encoded by these genes have been well documented by previous reports; these functions include xyloglucan biosynthesis by CslC [13,16], xyloglucan remodeling by XTH [5,12,17] and endoglucanase [18], xylan synthesis by GT 43 and GT 8 [19]. Recently, transcriptome profiling revealed that the genes involved in the organization of primary and secondary cell walls were highly expressed in the early elongating stage or in the sink-to-source transition of the leaf [20,21]. In the present study, we also observed that the biosynthesis and remodeling-related genes of xyloglucan were upregulated, specifically in sink-to-source transition. Given that xyloglucan endo-transglycolylase (XET) is directly involved in wall-loosening [22,23,24], our finding also supports that xyloglucan, which is a primary cell wall component, is also essential for wall organization in the sink-to-source transition of leaf blades of monocotyledonous plants, such as rice. Plant secondary cell wall biosynthesis is composed of two distinct biosynthetic pathways ((1) fatty acid-based polymers (suberin and cutin), and (2) phenylalanine-based polymers (lignin)) that differ from the stages of primary cell wall development. In our study, some of the genes encoding acyltransferases (ATs) and peroxidases (PODs), which are required to synthesize suberin, cutin and lignin, were noticeably upregulated during the source stage. Indeed, the genes observed were shown to be directly involved in the biosynthesis of a precursor of suberin and cutin via FARs [25,26], GPAT [27,28], BAHD acyltransferase [29,30] and cutin/suberin transport process involving plasma-membrane-localized ATP-binding cassette (ABC) transporters [31] in various plant organs, including leaves. One possible suggestion, derived from our findings, is that the active synthetic processes of secondary cell walls are much more dominant in the late developing stage of the whole process of leaf blade development. Developing leaf blades requires the import of photo-assimilates from mature leaves [5,32], which is similar to our observation (Figure 3 and Table 3). The leaf blade undergoing sink-to-source transition was more likely to display cell organization and autotrophy-related gene expression, and, on the basis of these observations, we identified the concentrations of some soluble carbohydrates in the sink-to-source transition and source stages, compared to the sink stage. Interestingly, it revealed that raffinose was also relative higher in the source stage compared to the sink stage and might be considered as an important photo-assimilate in carbon transport. The altered patterns in this study are supported by previous findings that the levels of monosaccharides, such as glucose, fructose and galactose, were the highest in young and elongating leaves, whereas sucrose was most abundant in older leaves [9,10,33]. Taken together, our findings show that cell wall organization and soluble carbohydrate abundance are closely associated with the development of the leaf blade; specifically, the development of sequential cell walls is strongly affected by the import of photo-assimilates from source tissues, a hypothesis that could be well supported by the review in [34].

Our survey suggests that the development of plant cell walls considered as a synchronized and an independent event is closely associated with an abundance of soluble carbohydrates in the developing leaf blade. Taken together, our results offer detailed insight into the interaction between cell wall organization and soluble carbohydrate abundance during rice leaf blade maturation.

## 4. Materials and Methods

### 4.1. Plant Materials and Sampling

Rice seeds (*Oryza sativa* L. cv. Saechucheong-byeo) after surface sterilization (2% sodium hypochlorite) were germinated for 3 days at 25 °C in darkness; the uniform seedlings were transplanted into plastic containers (20 cm × 10 cm × 5 cm) filled with commercial bed soil (Boonong Co., Ltd., Gyeongju, Korea) and grown in an environment-controlled growth chamber with a 14 h photoperiod, 25/20 °C (day/night) temperature, 60% relative humidity (RH), and 300~350 μmol quanta m^–2^ s^–1^ photosynthetic photo flux density (PPFD) at the top of the seedlings. The blades of the fifth leaves emerging from the sheaths of the fourth leaves were harvested and defined as sink (<0.5 cm of leaf blades, Day 1), sink-to-source (Day 3), and source stage (Day 5), as appropriate. The blades from 10~15 plants were harvested as a pool and were frozen in LN_2_ for transcriptomic and soluble carbohydrates analyses. Three biological replicates were taken from each stage (See Figure 1).

### 4.2. RNA Extraction, cDNA Synthesis and qRT-PCR

The total RNA from the leaf blades was extracted using a Total RNA Extraction Kit (Intron Biotech., Seongnam, Korea) according to the manufacturer’s instruction. The purity and concentration of the extracted RNA were estimated using NanoDrop (Thermo Fisher Scientific, Madison, WI, USA) and checked on a 1.2% agarose gel. A first-strand synthesis performed using a Maxime RT PreMix Kit with Oligo (dT) primers was used for cDNA synthesis from 1 μg of total RNA. The qRT-PCR was performed to analyze the relative gene expression of RNA using EvaGreen Q Master (LaboPass, Seoul, Korea) with a CFX Connect Optics Real-Time System (Bio-Rad, Hercules, CA, USA) according to the manufacturer’s instructions. A quantification method (2^–ΔΔCt^) was used and the variation in expression was estimated using three biological replicates. The rice actin gene was used as an internal control to normalize the data. The PCR conditions consisted of an initial denaturation step at 95 °C for 25 s, followed by 60 cycles of denaturation at 95 °C for 10 s, and annealing and extension at a melting temperature (Tm, °C) designated by each of the primer sets for 5 s (Table S1).

### 4.3. RNA-Seq Library Construction, Sequencing and DEGs Analysis

Sequencing was performed with each library to generate transcriptome sequences on an Illumina High-Seq 2500 platform, provided by a commercial service provider (Theragen Bio, Seongnam, Korea) [35]. Raw sequences in FASTQ format obtained from the Illumina platform were analyzed using publicly available tools. The low quality bases (Q < 15) was trimmed at both ends of sequence using customized program and the adapter was trimmed using Cutadapt [36]. The sequence was mapped to IRGSP-1.0 reference genome sequence using Bowtie [37] for short read mapping and TopHat to define exon-intron junctions [38]. Reference genome-based read assemblies were performed using Cufflinks and Cuffmerge. Expression levels of each transcript were expressed as kilobases per million fragments (FPKM) values calculated based on the number of mapped reads. All differential gene expressions (DEGs) were determined using Cufflinks program v.2.0.1 (http://cufflinks.cbcb.umd.edu; accessed on 15 January 2021) as described [39]. Each gene’s expression level was normalized using reads fragments per kilobase of exon per million mapped reads (RPKM). All DEGs were identified using differentially expressed genes(DEGs), and categorized to the gene ontology (GO) framework using DAVID bioinformatics resources (ver. 6.8) (https://david.ncifcrf.gov/tools.jsp accessed on 15 January 2021). To analyze the potential functions of rice proteins, genes were searched for in the Rice Annotation Project (RAP) databases, including the National Center for Biotechnology Information (NCBI) non-redundant, UniProt and the Kyoto Encyclopedia of Genes and Genomes (KEGG) pathway databases.

### 4.4. Soluble Carbohydrates Analysis

The extraction of polar metabolites from powdered samples (100 mg) was carried out by adding 1 mL of 2.5:1:1 (*v*/*v*/*v*) methanol:water:chloroform [40]. Ribitol (60 µL, 0.2 mg mL^–1^) was used as an internal standard (IS). Briefly, the polar-phased extracts were subjected to methoxime (MO)-derivatization and trimethylsilyl (TMS) etherification. The derivatized samples (1 µL) were quantified using an Agilent 7890A gas chromatograph (Agilent, Atlanta, GA, USA) coupled to a Pegasus high throughput time-of-flight (HT TOF) mass spectrometer (LECO, St. Joseph, MI, USA). The quantitation of all the analytes was based on the peak area ratio of each analyte relative to the peak area of the IS. See the S2 for the detail.

### 4.5. Statistics

The significance of the differences between the means was assessed using a *p*-value of < 0.05. All analyses were performed using PRISM 6 software (ver. 6.01, GraphPad Software Inc., San Diego, CA, USA).

## Figures and Tables

**Table 1:** Expression of primary cell wall organization-related genes by RNA-seq and qRT-PCR (mean ± S.D.).

GO Term	Gene ID	Gene	Short Description	Log_2_ FC	*p*-Value	Relative Upregulation
Cell wall organization	Os05g0123100	GT 43	Xylan synthesis	4.1	0.021	5.0 ± 0.6 ***
	Os10g0577500	XTH	Xyloglucan cleavage	6.0	0.035	5.3 ± 0.5 **
	Os06g0247900	Endoglucanase 16	Xyloglucan cleavage	4.7	0.008	1.6 ± 1.1 *
	Os09g0428000	CslC	Xyloglucan synthesis	3.9	0.039	1.7 ± 1.4
Polysaccharide biosynthetic process	Os03g0678800	GT 8	Xylan biosynthesis	9.1	0.002	4.1 ± 1.3 ***
	Os04g0530900	GT 8	Xylan biosynthesis	4.3	0.037	2.8 ± 0.9 **

An asterisk, *, ** and ***, means *p* < 0.05, 0.01 and 0.001 determined by LSD test. An actin was used as a control.

**Table 2:** Expression of secondary cell wall organization-related genes by RNA-seq and qRT-PCR (mean ± S.D.).

GO Term	Gene ID	Gene Name	Description	Log_2_ FC	*p*-Value	Relative Upregulation
Suberin and cutin biosynthesis	Os09g0567500	FAR	Fatty acyl reduction	16.7	0.022	1.4 ± 0.8 *
	Os03g0281900	ABC transporter	Wax/Cutin transport	19.7	0.048	4.1 ± 0.3 *
	Os11g0679700	GPAT 3	Suberin/Cutin fatty alcohol transfer	6.9	0.022	1.9 ± 0.5 *
	Os08g0562500	BAHD acyltransferase	BAHD acyl transfer	36.8	0.019	1.2 ± 0.9 *
H_2_O_2_ catabolism	Os02g0236600	POD	H_2_O_2_ peroxidation	47.2	0.049	3.1 ± 0.0 ***
	Os05g0499300	POD	H_2_O_2_ peroxidation	24.6	0.037	1.5 ± 0.2
	Os07g0677100	POD	H_2_O_2_ peroxidation	24.1	0.037	2.4 ± 0.0 *

An asterisk, * and ***, means *p* < 0.05 and 0.001 determined by LSD test. An actin was used as a control.

**Table 3:** Concentrations of selected soluble carbohydrates at the sink-to-source, source, and sink stages in the blades (mean ± S.D.).

Stage	Glucose	Fructose	Galactose	Sucrose	Raffinose
Sink	42.8 ± 1.7	13.3 ± 0.4	0.3 ± 0.1	180.0 ± 23.3	0.9 ± 0.1
Sink-to-source	15.6 ± 1.7	5.1 ± 0.7	0.1 ± 0.0	211.3 ± 57.8	0.9 ± 0.4
Source	26.0 ± 1.0	7.9 ± 0.4	0.4 ± 0.1	335.8 ± 66.0	2.8 ± 0.6

## Data Availability

The datasets analyzed during the current study are available from the corresponding author upon reasonable request.

## Supplementary Materials

The following are available online at https://www.mdpi.com/article/10.3390/plants10050823/s1, Table S1: Primer sequences used for qRT-PCR; Table S2: Alignment statistics for mapping of reads; Table S3: Library sizes and statistics information for RNA sequencing; Table S4: GO enrichment analysis of upregulated genes in libraries; S5: Extraction and analysis of polar metabolites.
