# Supplementary material for: Transcriptional Changes of Cell Wall Organization Genes and Soluble Carbohydrate Alteration during Leaf Blade Development of Rice Seedlings
Source: Plants (Basel). 2021 Apr 21;10(5):823. doi: 10.3390/plants10050823 (PMC8143110; doi:10.3390/plants10050823)
Supplement: Supplementary file 1 [file plants-10-00823-s001.zip › plants-1140240-supplementary.pptx]

## Slide 1
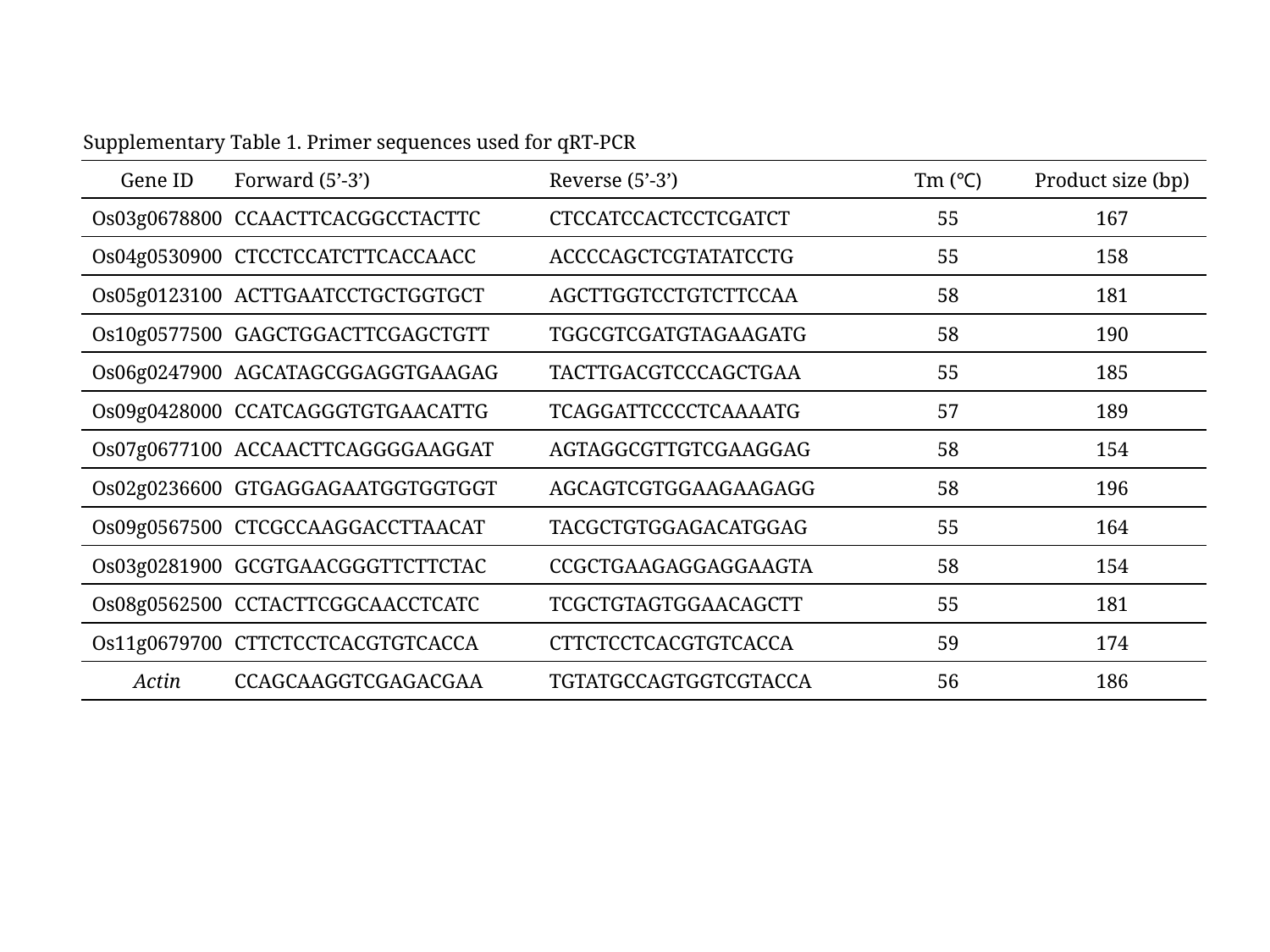

Supplementary Table 1. Primer sequences used for qRT-PCR
| Gene ID | Forward (5’-3’) | Reverse (5’-3’) | Tm (℃) | Product size (bp) |
| --- | --- | --- | --- | --- |
| Os03g0678800 | CCAACTTCACGGCCTACTTC | CTCCATCCACTCCTCGATCT | 55 | 167 |
| Os04g0530900 | CTCCTCCATCTTCACCAACC | ACCCCAGCTCGTATATCCTG | 55 | 158 |
| Os05g0123100 | ACTTGAATCCTGCTGGTGCT | AGCTTGGTCCTGTCTTCCAA | 58 | 181 |
| Os10g0577500 | GAGCTGGACTTCGAGCTGTT | TGGCGTCGATGTAGAAGATG | 58 | 190 |
| Os06g0247900 | AGCATAGCGGAGGTGAAGAG | TACTTGACGTCCCAGCTGAA | 55 | 185 |
| Os09g0428000 | CCATCAGGGTGTGAACATTG | TCAGGATTCCCCTCAAAATG | 57 | 189 |
| Os07g0677100 | ACCAACTTCAGGGGAAGGAT | AGTAGGCGTTGTCGAAGGAG | 58 | 154 |
| Os02g0236600 | GTGAGGAGAATGGTGGTGGT | AGCAGTCGTGGAAGAAGAGG | 58 | 196 |
| Os09g0567500 | CTCGCCAAGGACCTTAACAT | TACGCTGTGGAGACATGGAG | 55 | 164 |
| Os03g0281900 | GCGTGAACGGGTTCTTCTAC | CCGCTGAAGAGGAGGAAGTA | 58 | 154 |
| Os08g0562500 | CCTACTTCGGCAACCTCATC | TCGCTGTAGTGGAACAGCTT | 55 | 181 |
| Os11g0679700 | CTTCTCCTCACGTGTCACCA | CTTCTCCTCACGTGTCACCA | 59 | 174 |
| Actin | CCAGCAAGGTCGAGACGAA | TGTATGCCAGTGGTCGTACCA | 56 | 186 |

## Slide 2
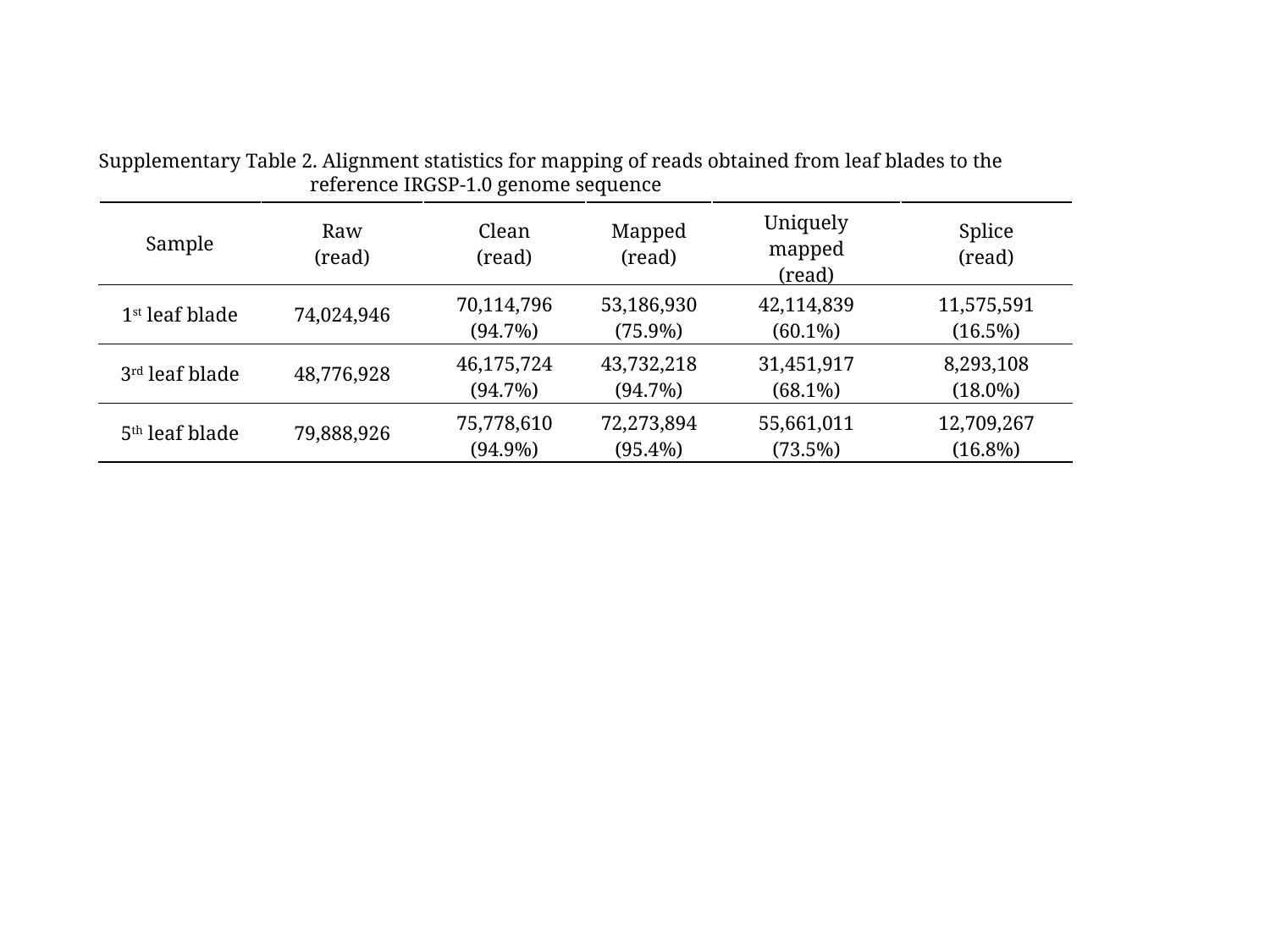

Supplementary Table 2. Alignment statistics for mapping of reads obtained from leaf blades to the reference IRGSP-1.0 genome sequence
| Sample | Raw (read) | Clean (read) | Mapped (read) | Uniquely mapped (read) | Splice (read) |
| --- | --- | --- | --- | --- | --- |
| 1st leaf blade | 74,024,946 | 70,114,796 (94.7%) | 53,186,930 (75.9%) | 42,114,839 (60.1%) | 11,575,591 (16.5%) |
| 3rd leaf blade | 48,776,928 | 46,175,724 (94.7%) | 43,732,218 (94.7%) | 31,451,917 (68.1%) | 8,293,108 (18.0%) |
| 5th leaf blade | 79,888,926 | 75,778,610 (94.9%) | 72,273,894 (95.4%) | 55,661,011 (73.5%) | 12,709,267 (16.8%) |

## Slide 3
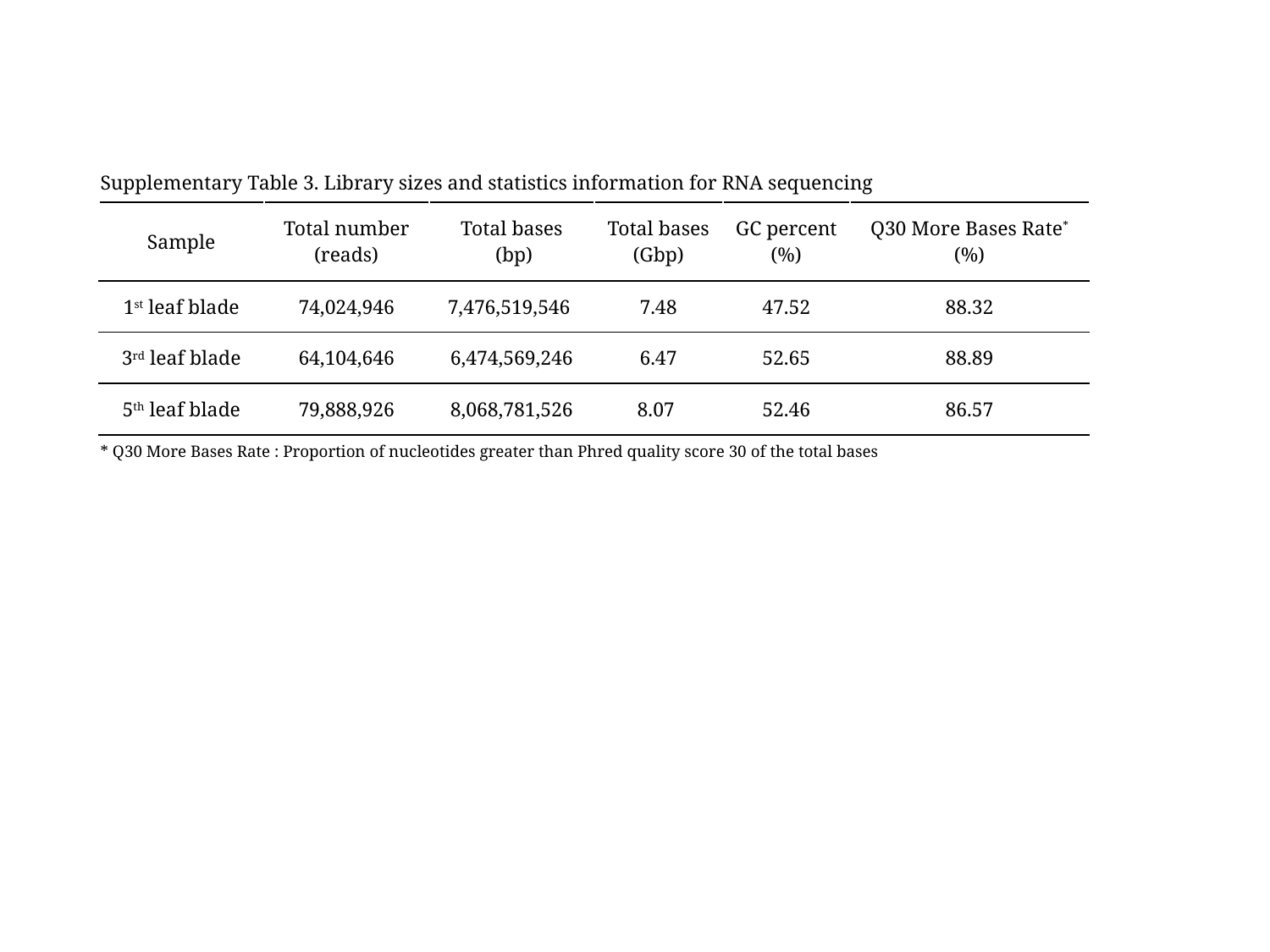

Supplementary Table 3. Library sizes and statistics information for RNA sequencing
| Sample | Total number (reads) | Total bases (bp) | Total bases (Gbp) | GC percent (%) | Q30 More Bases Rate\* (%) |
| --- | --- | --- | --- | --- | --- |
| 1st leaf blade | 74,024,946 | 7,476,519,546 | 7.48 | 47.52 | 88.32 |
| 3rd leaf blade | 64,104,646 | 6,474,569,246 | 6.47 | 52.65 | 88.89 |
| 5th leaf blade | 79,888,926 | 8,068,781,526 | 8.07 | 52.46 | 86.57 |
* Q30 More Bases Rate : Proportion of nucleotides greater than Phred quality score 30 of the total bases

## Slide 4
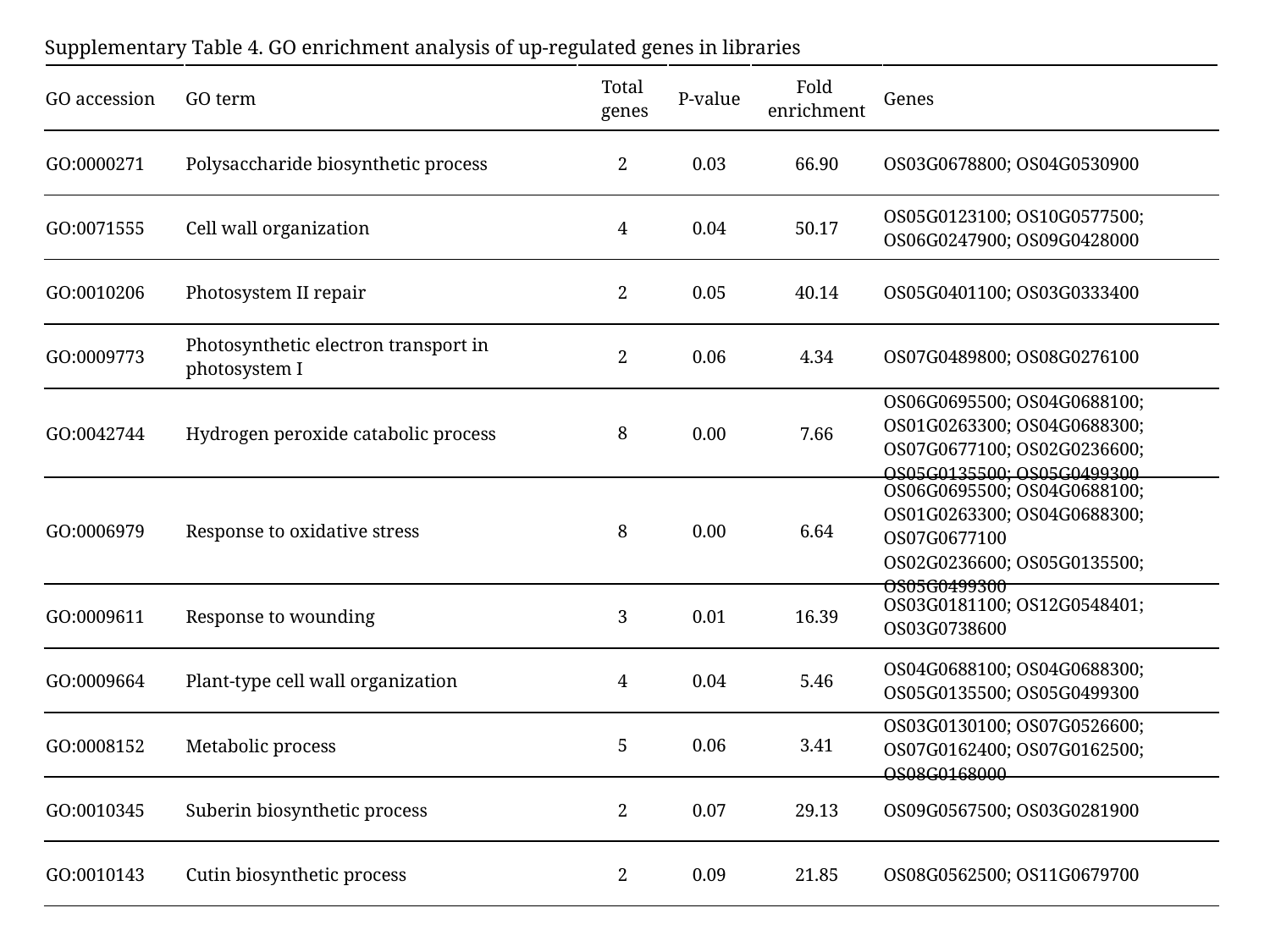

Supplementary Table 4. GO enrichment analysis of up-regulated genes in libraries
| GO accession | GO term | Total genes | P-value | Fold enrichment | Genes |
| --- | --- | --- | --- | --- | --- |
| GO:0000271 | Polysaccharide biosynthetic process | 2 | 0.03 | 66.90 | OS03G0678800; OS04G0530900 |
| GO:0071555 | Cell wall organization | 4 | 0.04 | 50.17 | OS05G0123100; OS10G0577500; OS06G0247900; OS09G0428000 |
| GO:0010206 | Photosystem II repair | 2 | 0.05 | 40.14 | OS05G0401100; OS03G0333400 |
| GO:0009773 | Photosynthetic electron transport in photosystem I | 2 | 0.06 | 4.34 | OS07G0489800; OS08G0276100 |
| GO:0042744 | Hydrogen peroxide catabolic process | 8 | 0.00 | 7.66 | OS06G0695500; OS04G0688100; OS01G0263300; OS04G0688300; OS07G0677100; OS02G0236600; OS05G0135500; OS05G0499300 |
| GO:0006979 | Response to oxidative stress | 8 | 0.00 | 6.64 | OS06G0695500; OS04G0688100; OS01G0263300; OS04G0688300; OS07G0677100 OS02G0236600; OS05G0135500; OS05G0499300 |
| GO:0009611 | Response to wounding | 3 | 0.01 | 16.39 | OS03G0181100; OS12G0548401; OS03G0738600 |
| GO:0009664 | Plant-type cell wall organization | 4 | 0.04 | 5.46 | OS04G0688100; OS04G0688300; OS05G0135500; OS05G0499300 |
| GO:0008152 | Metabolic process | 5 | 0.06 | 3.41 | OS03G0130100; OS07G0526600; OS07G0162400; OS07G0162500; OS08G0168000 |
| GO:0010345 | Suberin biosynthetic process | 2 | 0.07 | 29.13 | OS09G0567500; OS03G0281900 |
| GO:0010143 | Cutin biosynthetic process | 2 | 0.09 | 21.85 | OS08G0562500; OS11G0679700 |

## Slide 5
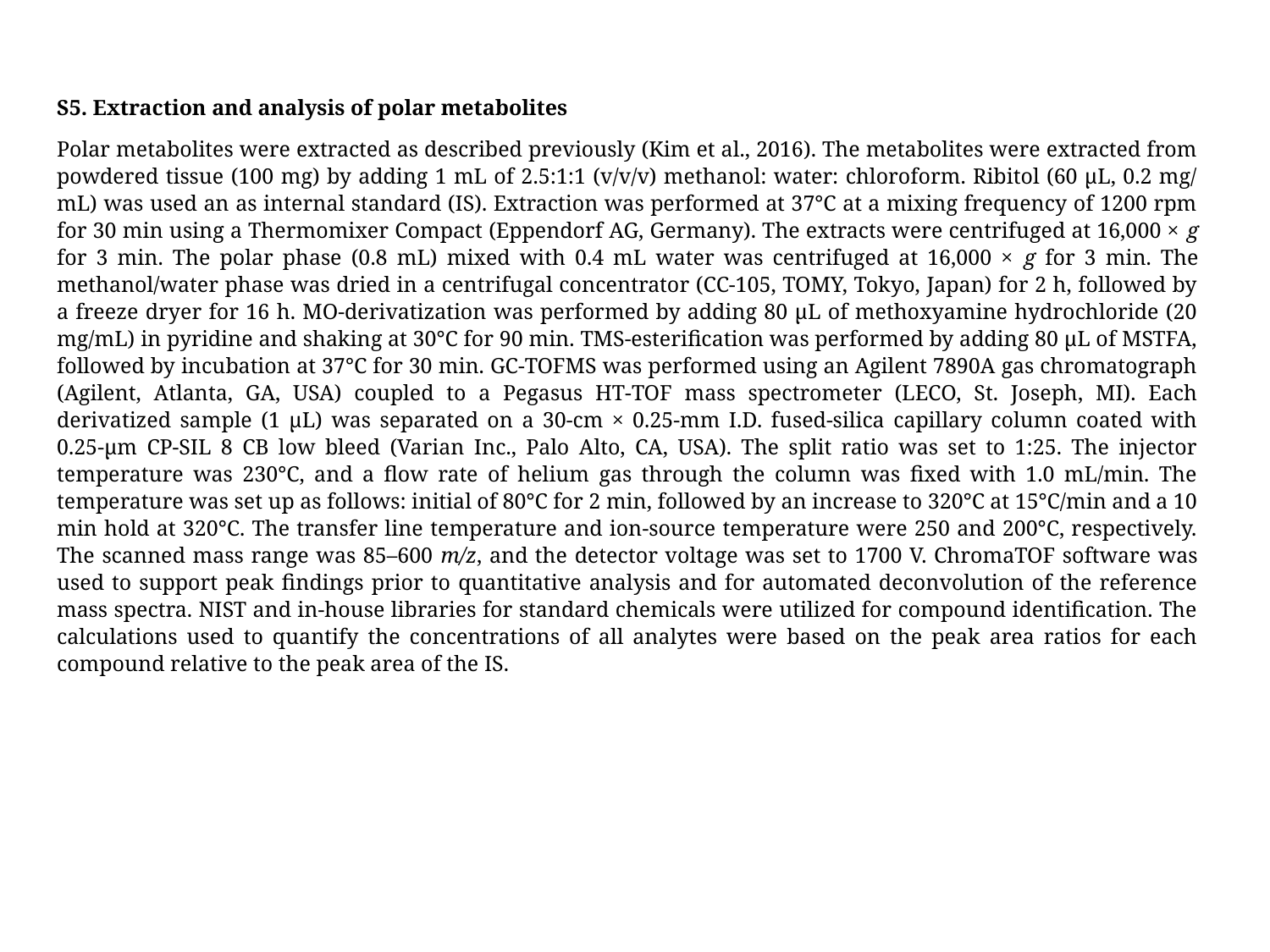

S5. Extraction and analysis of polar metabolites
Polar metabolites were extracted as described previously (Kim et al., 2016). The metabolites were extracted from powdered tissue (100 mg) by adding 1 mL of 2.5:1:1 (v/v/v) methanol: water: chloroform. Ribitol (60 µL, 0.2 mg/mL) was used an as internal standard (IS). Extraction was performed at 37°C at a mixing frequency of 1200 rpm for 30 min using a Thermomixer Compact (Eppendorf AG, Germany). The extracts were centrifuged at 16,000 × g for 3 min. The polar phase (0.8 mL) mixed with 0.4 mL water was centrifuged at 16,000 × g for 3 min. The methanol/water phase was dried in a centrifugal concentrator (CC-105, TOMY, Tokyo, Japan) for 2 h, followed by a freeze dryer for 16 h. MO-derivatization was performed by adding 80 μL of methoxyamine hydrochloride (20 mg/mL) in pyridine and shaking at 30°C for 90 min. TMS-esterification was performed by adding 80 μL of MSTFA, followed by incubation at 37°C for 30 min. GC-TOFMS was performed using an Agilent 7890A gas chromatograph (Agilent, Atlanta, GA, USA) coupled to a Pegasus HT-TOF mass spectrometer (LECO, St. Joseph, MI). Each derivatized sample (1 µL) was separated on a 30-cm × 0.25-mm I.D. fused-silica capillary column coated with 0.25-µm CP-SIL 8 CB low bleed (Varian Inc., Palo Alto, CA, USA). The split ratio was set to 1:25. The injector temperature was 230°C, and a flow rate of helium gas through the column was fixed with 1.0 mL/min. The temperature was set up as follows: initial of 80°C for 2 min, followed by an increase to 320°C at 15°C/min and a 10 min hold at 320°C. The transfer line temperature and ion-source temperature were 250 and 200°C, respectively. The scanned mass range was 85–600 m/z, and the detector voltage was set to 1700 V. ChromaTOF software was used to support peak findings prior to quantitative analysis and for automated deconvolution of the reference mass spectra. NIST and in-house libraries for standard chemicals were utilized for compound identification. The calculations used to quantify the concentrations of all analytes were based on the peak area ratios for each compound relative to the peak area of the IS.
